# “MURAL” model to predict bleeding from mural-based lesions in potential small bowel bleeding may improve diagnostic capability and decrease cost

**DOI:** 10.1097/MD.0000000000031989

**Published:** 2022-12-02

**Authors:** Julajak Limsrivilai, Thanaboon Chaemsupaphan, Sipawath Khamplod, Sitthipong Srisajjakul, Chayanis Kositamongkol, Pochamana Phisalprapa, Kochakon Maipang, Uayporn Kaosombatwattana, Nonthalee Pausawasdi, Phunchai Charatcharoenwitthaya, Somchai Leelakusolvong, Supot Pongprasobchai

**Affiliations:** a Division of Gastroenterology, Department of Medicine, Faculty of Medicine Siriraj Hospital, Mahidol University, Bangkok, Thailand; b Department of Radiology, Division of Diagnostic Radiology, Faculty of Medicine, Siriraj Hospital, Mahidol University, Bangkok, Thailand; c Division of Ambulatory Medicine, Department of Medicine, Faculty of Medicine Siriraj Hospital, Mahidol University, Bangkok, Thailand.

**Keywords:** cost effectiveness, computed tomography enterography, mural, small bowel bleeding, video capsule endoscopy

## Abstract

In potential small bowel bleeding, video capsule endoscopy (VCE) is excellent to detect mucosal lesions, while mural-based lesions are better detected by computed tomography enterography (CTE). A predictive tool to identify mural-based lesions should guide selecting investigations. In this retrospective study, we developed and validated the “MURAL” model based on logistic regression to predicts bleeding from mural-based lesions. Cost-effectiveness analysis comparing diagnostic strategy among VCE, CTE, and MURAL model was performed. Of 296 patients, 196 and 100 patients were randomly included in the derivative and validation cohorts, respectively. The MURAL model comprises 5 parameters: age, presence of atherosclerosis, chronic kidney disease, antiplatelet use, and serum albumin level. The area under the receiver operating characteristic curve was 0.778 and 0.821 for the derivative and validation cohorts, respectively. At a cutoff value of 24.2%, the model identified mural-based lesions with 70% sensitivity and 83% specificity in the validation cohort. Cost-effectiveness analysis revealed that application of the MURAL model demonstrated a comparable missed lesion rate but had a lower missed tumor rate, and lower cost compared to VCE strategy. The model for predicting mural-based lesions provide some guidance in investigative decision-making, which may improve diagnostic efficiency and reduce costs.

## 1. Introduction

Potential small bowel bleeding (PSBB), which was previously referred to as obscure gastrointestinal bleeding, is defined as gastrointestinal bleeding in which the bleeding source is undetectable by esophagogastroduodenoscopy and colonoscopy.^[1]^ Etiology can be categorized based on pathology into mucosal lesions, such as angiodysplasia, Dieulafoy’s lesion, and ulcer/inflammation, and mural-based lesions, such as small bowel mass and diverticulum.^[2]^ Investigations for PSBB include video capsule endoscopy (VCE), device-assisted enteroscopy (DAE), and radiographic examinations. The decision regarding which investigation to use depends on treatment purpose, availability, and cost. According to American College of Gastroenterology and American Society for Gastrointestinal Endoscopy guidelines, VCE should be performed first in clinically stable patients with PSBB given its high diagnostic yield and noninvasive nature.^[1,3]^ However, VCE could miss some lesions, particularly small bowel tumors, which were reported to be missed in up to 19% of cases.^[4]^ Computed tomography enterography (CTE), which has a higher sensitivity for detection of mural-based small bowel masses,^[5–9]^ is therefore recommended in those with negative VCE or in those with suspected small bowel tumor.^[1,3]^ Furthermore, even when a tumor is seen on VCE, CT images of the abdomen are usually acquired later to precisely locate and stage the tumor before surgery. In this situation, the choice of CTE as the first investigation would obviate the need for VCE, and this would reduce the cost of treatment. In addition to small bowel masses, CTE has been reported to detect Meckel’s diverticulum, which is another mural-based lesion, in PSBB patients with negative VCE findings.^[9,10]^

For the above reasons, if we can identify patients with PSBB who are likely to have mural-based small bowel lesions and allocate them to CTE rather than VCE, we may be able to lower the rate of missed lesions and reduce costs. Although some models to predict the types of small bowel lesions in PSBB have been reported, those models specifically predict the probability of bleeding from vascular or ulcerative lesions.^[11,12]^ A model to predict mural-based lesions has not yet been reported. Hence, our study aimed to identify parameters that potentially predict mural lesions, and then integrate those parameters into a model that can predict small bowel bleeding from mural-based lesions. We also analyzed the cost-effectiveness of our model compared to VCE and CTE.

## 2. Material and methods

### 2.1. Patient population

This retrospective cohort study was conducted at Siriraj Hospital, which is a university-based tertiary referral center that is located in Bangkok, Thailand. Patients who presented with PSBB, either overt or occult, during January 2008 to September 2017 were included. In patients with recurrent bleeding, only data for the first episode of bleeding was included. Patient data, including clinical information derived from medical records, laboratory data, imaging data, and procedures, were gathered from our center’s institutional database.

### 2.2. Model development

Bleeding lesions were classified as mural-based lesions if they were small bowel tumors or Meckel’s diverticulum. All diagnosed mural-based lesions were confirmed by either pathological specimens or Meckel’s scan. Those patients were randomly allocated into either the derivative (70%) or validation (30%) cohorts. Parameters that potentially predict bleeding from mural-based lesions were identified from analysis of data from the derivative cohort. The statistically significant parameters were integrated in a model using logistic regression analysis. The model was given the name “MURAL” model. The model was then validated in validation cohort. The model can be accessed via muralmodel.com.

### 2.3. Cost-effectiveness analysis

**Figure 1.**
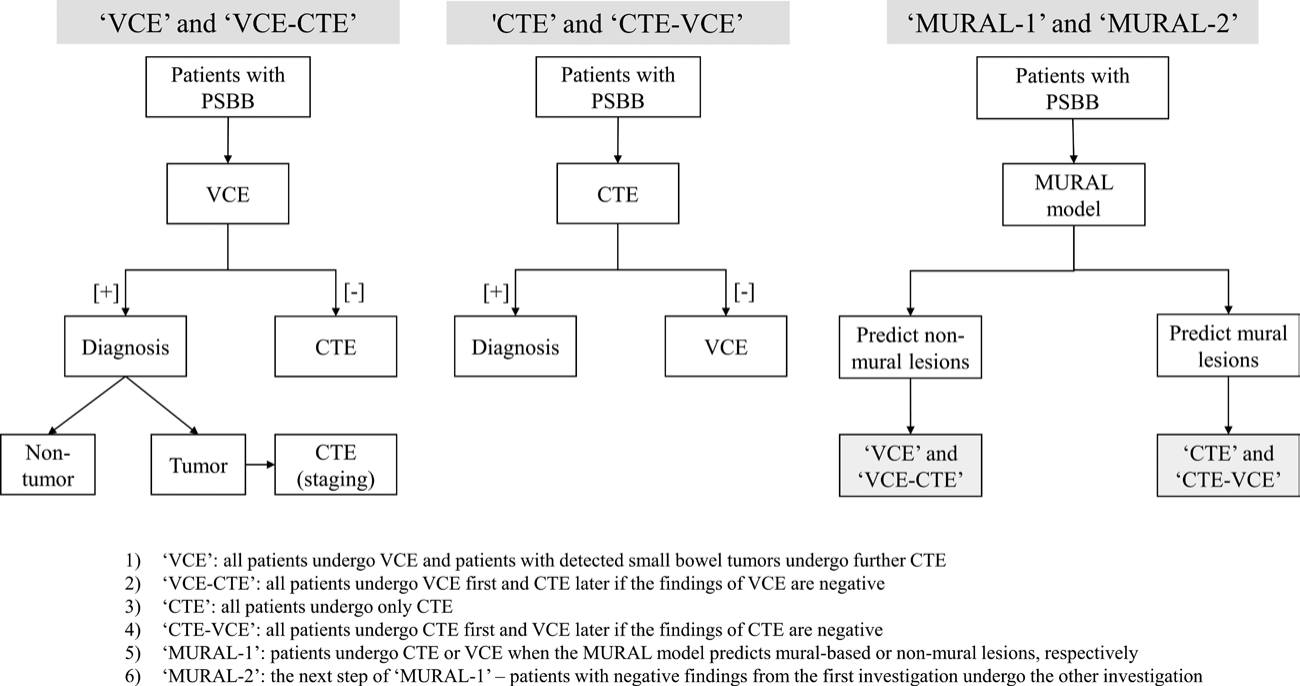
Strategy to investigate potential small bowel bleeding in cost-effectiveness analysis.

We compared 6 strategies using noninvasive investigations, including VCE, CTE, or both. These strategies were adapted from the recommendations of the American College of Gastroenterology, the American Society for Gastrointestinal Endoscopy, and the Japan Gastroenterological Endoscopy Society.^[1,3,13]^ The names and definitions of each strategy are, as follows: “VCE” - all patients underwent VCE, and patients with detected small bowel tumors underwent further CTE; “CTE” - all patients underwent only CTE; “MURAL-1” - the MURAL model was applied to all patients, and each patient underwent CTE or VCE when the model predicted mural-based or non-mural lesions, respectively; “VCE-CTE” - all patients underwent VCE first and CTE later if the findings of VCE were negative; “CTE-VCE” - all patients underwent CTE first and VCE later if the findings of CTE were negative; and “MURAL-2” (which was the next step after “MURAL-1”) - patients with negative findings of the first investigation underwent the other investigation. All strategies are shown in Figure 1.

In this analysis, we assumed that the lesions that caused small bowel bleeding comprised 3 major types of lesions, including angiodysplasia, ulcer/inflammation, and small bowel tumors. The prevalence of each lesion and the diagnostic performance of VCE and CTE in each type of lesion were obtained from previous studies, as shown in Table 1.^[9,14–19]^ The costs of VCE and CTE in the United States and Thailand were based on Center of Medicare and Medicaid service (www.cms.gov) and the costs in our hospital, respectively. All costs are reported in 2019 United States dollars (USD) (1 USD = 31 Thai baht), and in Thai baht. Outcomes, including the numbers of lesions that missed detection, the numbers of VCE and CTE needed, and the costs of investigations, were compared among all evaluated strategies.

**Table 1:** Prevalence of each lesion, and diagnostic performance of video capsule endoscopy and CT enterography in each type of lesion

Probability	Base-case estimate		Reported range		Reference
Prevalence of lesion			Eastern	Western	
•Angiodysplasia	50		26.7%	65.9%	^[14]^
•Ulcer/inflammation	30		37.6%	15.7%	^[14]^
•Tumor	20		26.4%	14.4%	^[14]^
VCE sensitivity/specificity	Sensitivity	Specificity	Sensitivity	Specificity	
•Angiodysplasia	90%	90%	60–100%	86–100%	^[9,15–17]^
•Ulcer/inflammation	85%	99%	81–100%	99–100%	^[9,15–17]^
•Tumor	55%	90%	33–70%	86–100%	^[9,15,16]^
CTE sensitivity/specificity	Sensitivity	Specificity	Sensitivity	Specificity	
•Angiodysplasia	20%	99%	0–80%	99–100%	^[9,15–19]^
•Ulcer/inflammation	30%	99%	9–66.7%	99–100%	^[9,15–19]^
•Tumor	90%	99%	70–100%	97–100%	^[9,15–19]^
Cost	United States*		Thailand*		
•VCE	1247 USD		42,000 Baht (1355 USD)		
•CTE	518 USD		15,400 Baht (497 USD)		

* The costs of VCE and CTE in the United States and Thailand were based on Center of Medicare and Medicaid service (www.cms.gov) and the costs in Siriraj Hospital, respectively.

CTE = computed tomography enterography, USD = United States Dollar, VCE = video capsule endoscopy.

The protocol of study was approved by the Siriraj Institutional Review Board of the Faculty of Medicine Siriraj Hospital, Mahidol University, Bangkok, Thailand on 26 September 2017 (COA no. 490/2560). The requirement to obtain informed consent was waived due to the retrospective nature of the study.

### 2.4. Statistical analysis

Continuous data are expressed as mean and standard deviation or median and range depending on data distribution. Categorical variables are given as frequency and percentage. Two group comparison was performed using independent *t*-test or Mann-Whitney U test for continuous variables, and chi-square test or Fisher’s exact test for categorical variables. Univariate and multivariate analysis to identify parameters associated with mural-based lesions was performed using logistic regression. Statistically significant parameters were then integrated into a logistic regression model. Regression coefficients were transformed into item scores, which were then added together to generate a total score that was used to calculate the probability of the presence of a mural-based lesion. A receiver operating characteristic curve was used to demonstrate model performance. The optimal cutoff value was determined by Youden Index. Sensitivity, specificity, and accuracy of the model were calculated. A *P*-value < 0.05 was considered statistically significant. SAS Statistics software (SAS, Inc., Cary, North Carolina) was used for all statistical analyses.

For cost-effectiveness analysis, outcomes including the numbers of lesions that missed detection, the numbers of VCE and CTE needed, and the costs of investigations were calculated based on reported prevalence and diagnostic performance using Microsoft Excel.

## 3. Results

Four hundred and 16 patients (50 mural, 366 non-mural) were identified. The mean age of patients was 64.6 ± 16.3 years, and 210 (50.6%) were male. Two hundred and 64 (63.5%) presented with overt bleeding. The characteristics and final diagnoses of patients are shown in Table 2. One hundred and 20 patients lacked some laboratory data and were excluded. The remaining 296 patients were included in the analysis. The clinical characteristics and laboratory data between the cohort of total patients and the cohort of patients with complete clinical and laboratory data were comparable, as shown in Table 2. To develop the model, 196 (34 mural and 162 non-mural lesions) and 100 (10 mural and 90 non-mural lesions) patients were separated into the derivative cohort and the validation cohort, respectively.

**Table 2:** Characteristics and final diagnoses of 416 patients in the total cohort, and in 296 patients with complete laboratory tests.

Parameters	Total cohort (n = 416)	Cohort with complete laboratory tests (n = 296)
Age (mean ± SD)	64.6 ± 16.3	66.5 ± 15.0
Male gender	210 (50.6%)	147 (49.7%)
Diabetes mellitus	128 (30.8%)	92 (31.1%)
Large vessel atherosclerotic disease	135 (32.5%)	107 (36.2%)
Chronic kidney disease	116 (27.9%) Hemodialysis 37 (8.9%)	97 (32.8%) Hemodialysis 31 (10.5%)
Chronic liver disease	49 (11.8%)	4 (9.1%)
Immunocompromised status	16 (4.4%)	14 (4.7%)
History of cancer	47 (11.3%)	41 (13.8%)
NSAIDs	25 (6.0%)	18 (6.1%)
Antiplatelets	133 (32.0%)	97 (32.8%)
Anticoagulant	55 (13.2%)	42 (14.2%)
Overt bleeding	264 (63.5%)	200 (67.6%)
Abdominal pain	15 (3.6%)	12 (4.1%)
Weight loss	31 (7.5%)	25 (8.5%)
Hemoglobin level (g/dL)	7.84 ± 2.41	7.3 ± 2.1
Albumin level (mg/dL)	3.41 ± 0.74	3.41 ± 0.74
**Final diagnosis**		
Ulcers/inflammation	80 (19.2%)	63 (21.3%)
Angiodysplasia	71 (17.1%)	58 (19.6%)
Tumor	43 (10.3%)	40 (13.5%)
Dieulafoy’s lesion	9 (2.1%)	7 (3.4%)
Diverticulum	7 (1.7%)	4 (1.4%)
Other small bowel bleeding	3 (0.7%)	3 (1.0%)
Non-small bowel bleeding	55 (13.2%)	36 (12.2%)
Uncertain diagnosis	148 (35.6%)	85 (28.7%)

### 3.1. Model development and validation

**Figure 2.**
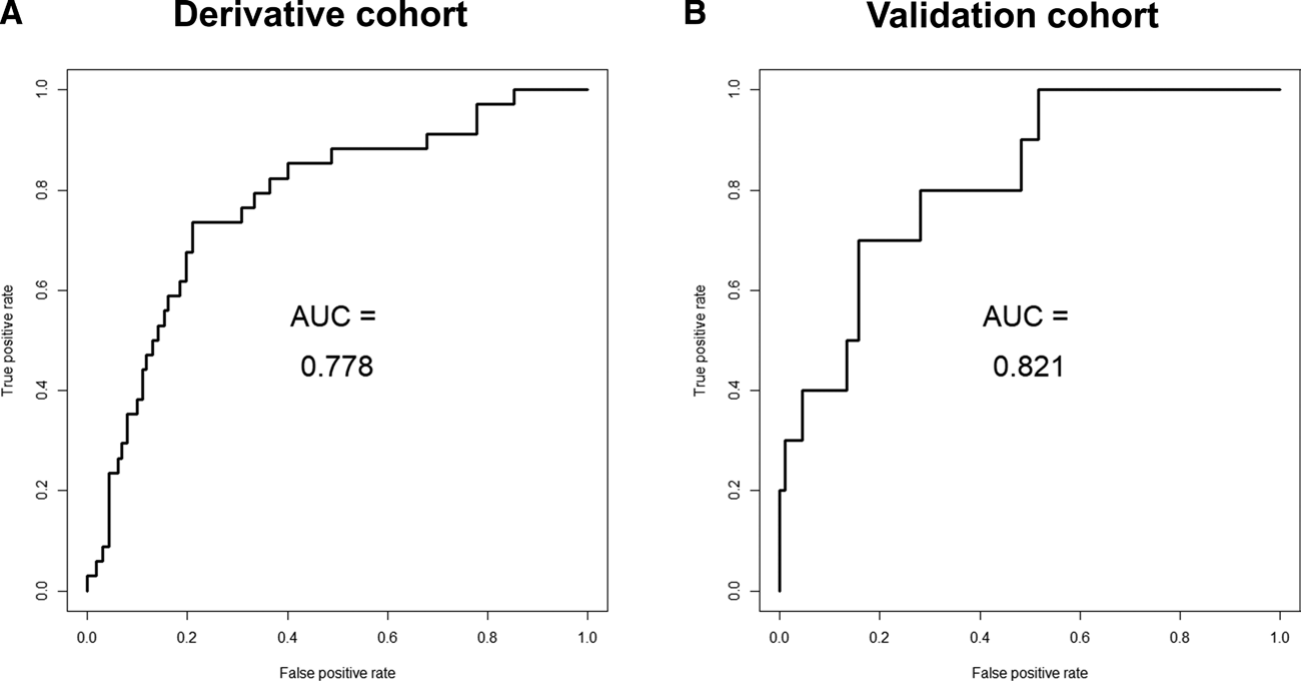
Receiver operating characteristic curve of the MURAL model when applied to a derivative cohort (Figure 2A) and a validation cohort (Figure 2B).


In the derivative cohort (Table 3), univariate analysis revealed 5 statistically significant parameters that predict the presence of mural-based lesions. These parameters included age (odds ratio [OR]: 0.97, 95% confidence interval [CI]: 0.95–0.99), presence of large vessel atherosclerotic diseases (OR: 0.15, 95% CI: 0.05–0.52), presence of chronic kidney disease (CKD) (OR: 0.24, 95% CI: 0.08–0.71), use of antiplatelets (OR: 0.17, 95% CI: 0.05–0.59), and serum albumin level (OR: 2.82, 95% CI 1.55–5.13). However, only serum albumin remained statistically significant in multivariate analysis with an OR of 2.37 (95% CI: 1.30–4.34). The model including only albumin level showed an area under the receiver operating characteristic curve (AUROC) of 0.683. Since the other significant parameters in univariate analysis were clinically meaningful, they were added into the model, and they were found to improve model performance. The AUROC of the model including all 5 significant parameters was 0.778 (95% CI: 0.69–0.865), as shown in Figure 2A. The formula of the logistic regression model to calculate the probability of mural-based lesion is shown below.

**Table 3:** Univariate and multivariate analysis for factors that predict mural lesions in training cohort.

Parameters			Univariate analysis		Multivariate analysis	
	Mural (n = 34)	Non-mural (n = 162)	Odds ratio (95% CI)	*P*-value	Odds ratio (95%CI)	*P*-value
Age (mean ± SD)	57.7 ± 13.0	65.3 ± 15.4	**0.97 (0.95–0.99**)	**.010**	0.99 (0.97–1.02)	.635
Male gender	21 (61.8%)	78 (48.2%)	1.74 (0.82–3.71)	.152		
Diabetes mellitus	6 (17.7%)	52 (32.1%)	0.45 (0.18–1.16)	.100		
Large vessel atherosclerotic disease	3 (8.8%)	63 (38.9%)	**0.15 (0.05–0.52**)	**.003**	0.35 (0.78–1.58)	.171
Chronic kidney disease	4 (11.8%)	58 (35.8%)	**0.24 (0.08–0.71**)	**.010**	0.54 (0.16–1.82)	.319
Chronic liver disease	4 (11.8%)	30 (18.5%)	0.59 (0.19–1.79)	.349		
Immunocompromised status	0	11 (6.8%)	cannot calculate	.968		
History of cancer	6 (17.7)	22 (13.6)	1.36 (0.51–3.67)	.539		
NSAIDs	3 (8.8%)	8 (4.9%)	1.86 (0.47–7.42)	.378		
Antiplatelets	3 (8.8%)	58 (35.8%)	**0.17 (0.05–0.59**)	**.005**	0.44 (0.10–1.87)	.265
Anticoagulant	2 (5.9%)	26 (16.1%)	0.33 (0.07–1.45)	.141		
Overt bleeding	22 (64.7%)	107 (66.1%)	0.94 (0.43–2.04)	.881		
Abdominal pain	1 (2.9%)	7 (4.3%)	0.67 (0.08–5.64)	.714		
Weight loss	5 (14.7%)	10 (6.2%)	2.62 (0.83–8.23)	.099		
Hemoglobin (g/dL)	6.92 ± 1.94	7.35 ± 2.21	0.91 (0.73–1.12)	.373		
Albumin (mg/dL)	3.87 ± 0.71	3.35 ± 0.74	**2.82 (1.55–5.13**)	**<.001**	**2.37 (1.30–4.34**)	**.005**

$$\begin{array}{l} P=100*\;\frac{1}{1+e^{-(\begin{array}{c} -3.7207-0.00696*age\\ -1.0531*atherosclerosis-0.6161*CKD\\ -0.8276*antiplatelet+0.8627*albumin \end{array})}} \end{array}$$

P = calculated probability of mural-based lesion

age; actual age in year, atherosclerosis; 1 = presence, 0 = absence, CKD; 1 = presence, 0 = absence, antiplatelet; 1 = use, 0 = not use, albumin; actual level in g/dL

The model can be accessed in muralmodel.com. The optimal cutoff value was the calculated probability of mural-based lesion of 24.2%. At this cutoff value, the sensitivity, specificity, and accuracy of the model to predict the presence of mural-based lesion was 73.5%, 79.0%, and 78.1%, respectively, in the derivative cohort. The model was then applied to the validation cohort, and it showed an AUROC of 0.821 (Fig. 2B). At the same cutoff value, the sensitivity, specificity, and accuracy were 70%, 83%, and 81%, respectively.

### 3.2. Cost-effectiveness analysis

The numbers of missed lesions, numbers of total investigations, and total costs of 6 strategies when they were applied to 100 patients in whom presumably 50% had angiodysplasia, 30% had inflammatory lesions, and 20% had tumors are shown in Table 4. Among the strategies that used only 1 investigation, the VCE strategy missed least, with only 18 of 100 lesions being missed. However, tumors were missed in 9 of 20 patients (45%), and its cost was most expensive. CTE missed the least number of tumors and costed the least; however, its missed lesion rate was the highest. MURAL-1 missed 24 lesions, which is 6 more than the VCE strategy. However, tumors were missed in only 4 patients, and the model decreased the cost of care by about 261 USD (8091 Thai baht) per patient when compared to the VCE strategy.

**Table 4:** Numbers of missed lesions, investigations, and cost of each strategy (values are shown per 100 patients unless otherwise specified).

	VCE	CTE	MURAL-1	VCE-CTE	CTE-VCE	MURAL-2
Total missed lesions	18	62	24	8	8	8
•Missed angiodysplasia	5/50 (10%)	40/50 (80%)	12/50 (24%)	4/50 (8%)	4/50 (8%)	4/50 (8%)
•Missed ulcers	4/30 (13.3%)	21/30 (70%)	8/30 (26.7%)	3/30 (10%)	3/30 (10%)	3/30 (10%)
•Missed tumors	9/20 (45%)	2/20 (10%)	4/20 (20%)	1/20 (5%)	1/20 (5%)	1/20 (5%)
Total investigations	119	100	113	137	162	133
•VCE	100	0	69	100	62	83
•CTE	19	100	44	37	100	50
Cost per patient						
•USD	1345	518	1084	1436	1296	1289
•Baht (USD)	44,926 (1449)	15,400 (497)	35,591 (1148)	47,629 (1536)	41,604 (1342)	42,394 (1368)

CTE = computed tomography enterography, USD = United States Dollar, VCE, video capsule endoscopy.

* The costs of VCE and CTE in the United States and Thailand were based on Center of Medicare and Medicaid service (www.cms.gov) and the costs in Siriraj Hospital, respectively.

When the 2^nd^ investigation was added, all 3 strategies detected lesions equally. The cost was highest in the VCE-CTE strategy, while the costs of the other 2 strategies were comparable. The numbers of investigations performed were most in the CTE-VCE strategy, in which 162 investigations (100 CTE and 62 VCE) were performed. The total numbers of investigations between the VCE-CTE and MURAL-2 strategy were comparable.

## 4. Discussion

**Figure 3.**
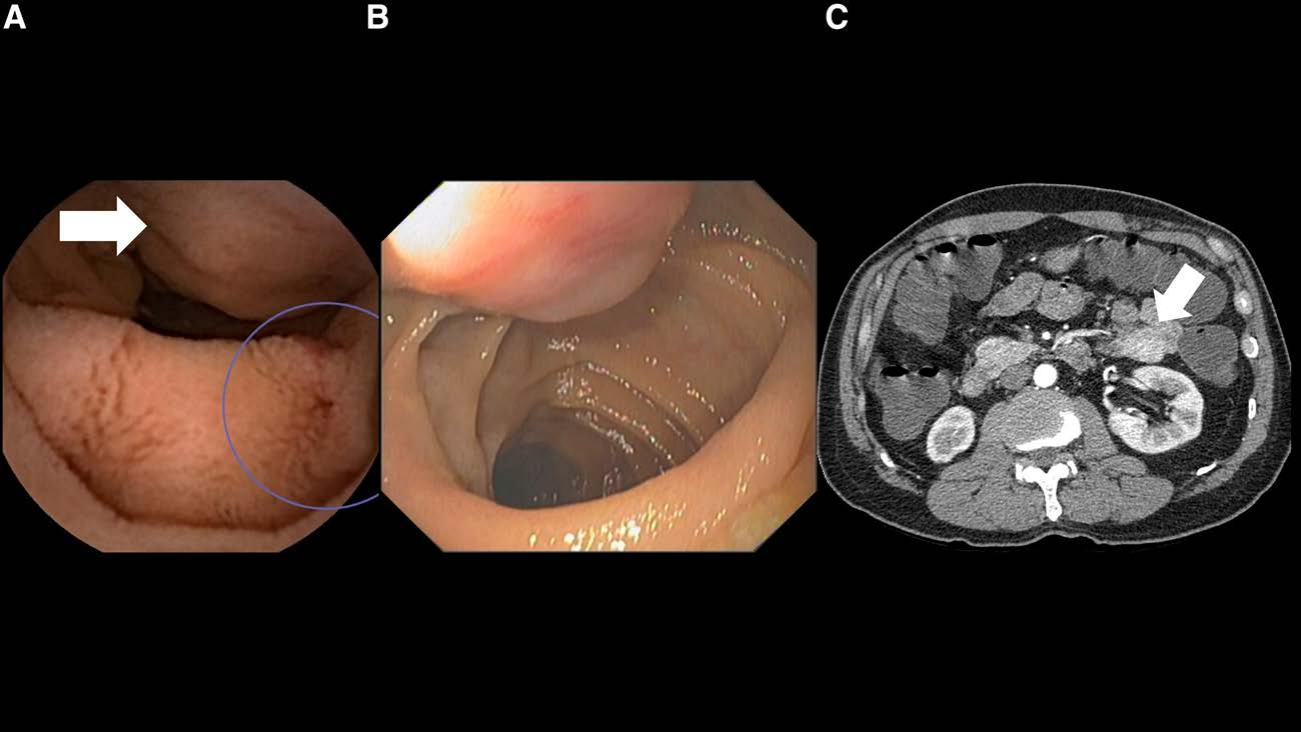
In Figure 3A, video capsule endoscopy (VCE) incorrectly reported an angiodysplasia at jejunum as a source of bleeding (purplish circle) but missed a mass beside (white arrow). The mass was discovered by balloon-assisted enteroscopy as shown in Figure 3B. CT abdomen was performed for preoperative assessment and showed a well circumscribed submucosal mass arising from collapsed proximal jejunum. The mass showed homogeneous hypervascular enhancement in Figure 3C. Surgery was performed and pathologically confirmed a gastrointestinal stromal tumor. CTE = computed tomography enterography, VCE = video capsule endoscopy.

**Figure 4.**
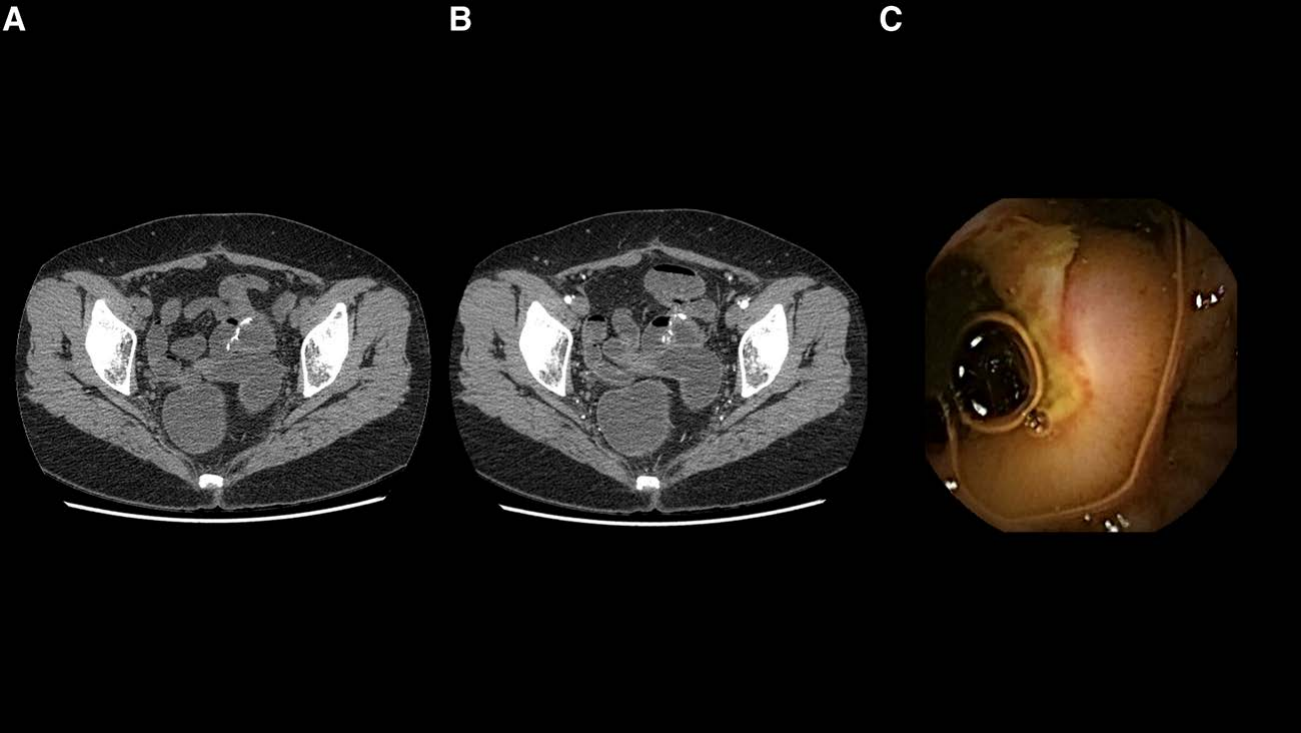
Figure 4A. Axial noncontrast enhanced CT shows patency of ileoileal anastomosis with the presence of high-density surgical materials. Figure 4B. Axial contrast enhanced CT shows no definite abnormal enhancement at anastomosis. Figure 4C. VCE showed a large clean-base ulcer at the anastomosis. CTE = computed tomography enterography, VCE = video capsule endoscopy.

Despite the development of many small bowel investigative tools, potential small bowel bleeding remains problematic. The main 3 investigative modalities include VCE, DAE, and imaging, and each of these investigations has its own advantages and disadvantages. The advantages of VCE include its ability to examine throughout the small bowel, its reasonably high diagnostic yield, and its non-invasiveness nature. However, it has some limitations. First, it lacks the ability to provide therapy to stop bleeding. This is particularly important if the bleeding is from vascular lesions, such as angiodysplasia and Dieulafoy’s lesion. Early intervention with DAE rather than delaying 1 day for VCE may increase the opportunity to find lesions during active bleeding, which would facilitate earlier management and prevent further or recurrent bleeding.^[20]^ Second, VCE can miss some lesions. The lesions that are relatively frequently missed are submucosal tumors because they look quite similar to innocent bulges as shown in Figure 3.^[21]^ Single lesions, such as a small bowel tumor or Meckel’s diverticulum, are also at higher risk for being missed by VCE. To detect these types of lesions, particularly small bowel submucosal tumors, CTE performs better.^[15,16,22–24]^ Despite the high diagnostic yield of DAE and its ability to provide therapeutic intervention, it is both invasive and time-consuming, which limits its use as a 1^st^ line investigation. Regarding CTE, although it is excellent in diagnosis of small bowel tumors, its use is limited due to its lower diagnostic yield compared to other investigations since the majority of bleeding lesions are mucosal lesions.^[25]^ Figure 4 showed an anastomotic ulcer which was observed in VCE but not in CTE. Radiation exposure in CTE is another risk that needs to be weighed against it benefit. The variety of strengths and weaknesses of the described diagnostic modalities suggests that the strategy to investigate PSBB should be individualized depending on the type of lesion that is suspected.

Predictive models for vascular lesions have recently been published.^[11,12]^ These models may help to triage patients with active and ongoing overt bleeding to undergo DAE if vascular lesion is suspected. However, to the best of our knowledge, a model to predict mural-based lesions, which could help to select patients with PSBB to be initially investigated with CTE, has not been published. Our study developed a model based on 196 patients with PSBB, and validated the model in another cohort of 100 patients. The significant variables from univariate analysis included age, presence of large vessel atherosclerotic disease, presence of CKD, use of antiplatelet, and albumin level. Younger age and higher level of serum albumin predicted mural-based lesions, while the others predicted non-mural lesions. Small bowel tumors and Meckel’s diverticulum, which account for the majority of mural-based lesions, are common in patients aged younger than 55 years.^[26,27]^ In contrast, intestinal angiodysplasia has reportedly increased in old age, chronic kidney disease, and atherosclerosis.^[12,28]^ The proposed pathogenesis is that these conditions cause vascular compromise, which leads to intestinal wall hypoxia and eventually stimulates angiogenetic factors.^[29]^ Antiplatelet agents can cause small bowel mucosal injuries and bleeding.^[30]^ Lower serum albumin levels could be the result of either protein loss from small bowel ulcers or may reflect poor nutritional status, which may potentiate the risk of small bowel injury from aspirin or NSAIDs. Serum albumin level was reported to be lower in severe NSAID-induced small intestinal damage when compared to no or mild damage.^[31]^ Even though only serum albumin was significant in multivariate analysis, we decided to include all significant parameters from univariate analysis in our “MURAL” model given their clinical significance based on reported associations with presence of either mural or non-mural lesions. Furthermore, the model including all factors performed better than the model including only serum albumin level. The model was applied to the validation cohort and showed similar performance.

For clinical application, we compared VCE, CTE, or both for investigating patients with PSBB in cost-effectiveness analysis. We found that each evaluated strategy had its own set of strengths and limitations. Some of the issues that should be considered when deciding upon an investigation include: diagnostic performance, including missed lesion rate and missed tumor rate; cost; risk of radiation exposure; and availability of the investigation. Among the strategies using only 1 investigation, VCE showed the best diagnostic yield when compared to other strategies. Furthermore, VCE confers the lowest risk of radiation exposure, since only those thought to need CTE undergo CTE. However, it is costly and can miss up to 19% of tumors. Another limitation is that until an artificial intelligence system can be developed to read VCE, an expert interpreter is required, and this level of skill is not available in all centers.

The strategy that used only CTE had the highest missed lesion rate (62%). This rate was considerably higher than the 18% and 23% rates for the VCE and MURAL-1 strategies, respectively. However, CTE missed the least number of tumors (2%). It was also the least expensive, and CTE may be more available than VCE in many centers. However, many patients undergoing CTE would be unnecessarily exposed to radiation.

Regarding the MURAL-1 strategy, although the missed lesion rate was higher than that in the VCE strategy, the difference was minimal. Furthermore, the missed tumor rate was 50% lower in MURAL-1 than in VCE. Other important benefits of MURAL-1 are that the cost was found to be 25% lower than VCE, and radiation exposure was 50% < CTE. Regarding appropriate fit of strategy with setting, VCE strategy may be more suitable in countries with high income and a lower number of tumors, whereas CTE strategy may be more suitable in low income countries with a high prevalence of small bowel tumors. The MURAL-1 strategy seems to fit somewhere between those 2 demographic and clinical settings. The “MURAL” model may help to decrease cost and avoid unnecessary radiation exposure while maintaining an acceptably high level of diagnostic performance.

The strength of this study is that it is the 1^st^ to report a model to predict mural-based lesions in patients with PSBB, which could help physicians to better select proper investigations in clinical practice. Moreover, this study also reports both the 1^st^ cost-effectiveness analysis comparing VCE and CTE, and the potential benefits and risks of each investigative strategy. However, this study also has some limitations. First, because of its retrospective design, many patients were excluded due to incomplete data, which resulted in a limited number of patients in both training and validation cohort. This reduced the power of the model to predict mural lesions. Second, in cost-effectiveness analysis we did not include Meckel’s diverticulum due to the relatively limited reported diagnostic yield of VCE and CTE for detecting Meckel’s diverticulum when compared to other lesions. However, the prevalence of Meckel’s diverticulum was reported to be uncommon compared to other types of lesions^[14]^; therefore, our omission of Meckel’s diverticulum should not exert significant influence on our results. Third, unlike previous cost-effectiveness studies,^[32,33]^ we did not include costs and risk of interventional treatment, such as endoscopy or surgery, in our analysis. Alternatively, we assumed that any lesions detected would be treated similarly regardless of strategy. Therefore, we focused only on diagnostic processes in our analysis.

In conclusion, the “MURAL” model for predicting mural-based lesions developed and validated in this study provides some guidance in investigative decision-making, which may improve diagnostic efficiency and reduce costs in patients with PSBB.

## Acknowledgements

The authors gratefully acknowledge Asst. Prof Kevin P. Jones, Medical Research Manuscript Editor, Siriraj Medical Research Center (SiMR), Faculty of Medicine Siriraj Hospital, Mahidol University for language editing.

## Author contributions

**Conceptualization:** Julajak Limsrivilai.

**Data curation:** Thanaboon Chaemsupaphan, Sipawath Khamplod.

**Formal analysis:** Julajak Limsrivilai, Chayanis Kositamongkol, Pochamana Phisalprapa.

**Investigation:** Julajak Limsrivilai.

**Methodology:** Julajak Limsrivilai.

**Project administration:** Julajak Limsrivilai.

**Resources:** Julajak Limsrivilai.

**Supervision:** Phunchai Charatcharoenwitthaya.

**Validation:** Julajak Limsrivilai.

**Visualization:** Julajak Limsrivilai.

**Writing – original draft:** Thanaboon Chaemsupaphan, Kochakon Maipang.

**Writing – review & editing:** Julajak Limsrivilai, Sitthipong Srisajjakul, Uayporn Kaosombatwattana, Nonthalee Pausawasdi, Phunchai Charatcharoenwitthaya, Supot Pongprasobchai.
